# Axis Orbit Recognition of the Hydropower Unit Based on Feature Combination and Feature Selection

**DOI:** 10.3390/s23062895

**Published:** 2023-03-07

**Authors:** Wushuang Liu, Yang Zheng, Xuan Zhou, Qijuan Chen

**Affiliations:** School of Power and Mechanical Engineering, Wuhan University, Wuhan 430072, China

**Keywords:** hydropower unit, axis orbit, feature combination, feature selection

## Abstract

Axis-orbit recognition is an essential means for the fault diagnosis of hydropower units. An axis-orbit recognition method based on feature combination and feature selection is proposed, aiming to solve the problems of the low recognition accuracy, poor robustness, and low efficiency of existing axis-orbit recognition methods. First, various contour, moment, and geometric features of axis orbit samples are extracted from the original data and combined into a multidimensional feature set; then, Random Forest (RF)-Fisher feature selection is applied to realize feature dimensionality reduction; and finally, the selected features are set as the input of the support vector machine (SVM), which is optimized by the gravitational search algorithm (GSA) for axis-orbit recognition. The analytical results show that the proposed method has high recognition efficiency and good robustness while maintaining high accuracy for axis-orbit recognition.

## 1. Introduction

As the largest renewable energy source in the world, hydropower has played an irreplaceable leading role in global energy conservation and emission reduction for decades [1]. As the core equipment of the energy conversion, hydropower units play a key role in the power systems, such as peak and frequency regulation and emergency standby [2]. In recent years, with the development of mechanical manufacturing technology and the high-speed construction of large hydropower and pumped storage power plants, hydropower units have shown development trends of large capacity, complex structure, and intelligent monitoring [3]. These characteristics will lead to some adverse effects on the stable operation of power plants, such as the possibility of failure during unit operation and the diversity of fault types. Once a fault occurs, it may cause damage to the normal operation of the unit, impact on the voltage and frequency stability of the power grid, and cause severe safety accidents and heavy economic losses [4].

Vibration is the most typical fault phenomenon in the process of unit operation. The existing research shows that about 80% of the faults are directly related to the vibration signal of the unit [5], and there is rich fault feature information in the vibration signal of the unit for data mining. Therefore, it has become the mainstream of unit fault diagnosis to adopt effective signal processing and feature extraction to obtain the key fault features contained in the vibration signal, and then establish a reasonable diagnostic model to identify the fault type and degree [6]. The axis orbit is synthesized by multiple vibration signals of measuring points in X and Y directions and can display the rotor trajectory intuitively. It is often used as an important indicator for the fault detection of hydropower units [3,7].

Traditional signal analysis mainly includes time-domain and frequency-domain analysis methods, which describe the equipment status information contained in the signal by extracting the time-domain statistical characteristics, such as peak-to-peak value and effective value of the signal [8], and the frequency-domain energy characteristics, such as the composition and size of frequency components [9], respectively. However, these two methods are vulnerable to changes in working conditions and interference noise and have greater limitations on the analysis of non-stationary signals [10]. Due to the complexity of the influencing factors of the non-stationary and nonlinear vibration signals of hydropower units, including hydraulic, mechanical, electromagnetic and other aspects, it is difficult to achieve satisfactory diagnosis results. Under this background, the time-frequency analysis methods of non-stationary signals represented by short-time Fourier transform [11], wavelet transform [12], empirical mode decomposition [13], and variational mode decomposition [14] are widely used in the field of the fault diagnosis of hydropower units.

The fault diagnosis of hydropower units is a mapping from the data space or feature space of monitoring signals to the fault space of units. At present, most of the fault-diagnosis methods of hydropower units applied in power stations still rely on the power spectrum and phase spectrum of the measured signals to identify fault states manually [15,16]. This conventional fault-diagnosis method with high human cost is highly dependent on expert knowledge and operation and maintenance experience, which is difficult to meet the needs of intelligent hydropower station construction. Therefore, it is urgent to carry out research on advanced fault-diagnosis methods for hydropower units, and integrate the operation and maintenance of hydropower units, signal processing, artificial intelligence and other disciplines, so as to replace the traditional fault-diagnosis methods based on reasoning and expert knowledge and improve the intelligent level of fault-diagnosis models. With the rise of machine learning research, more and more artificial intelligence technologies are widely used in the fault diagnosis of hydropower units to improve the accuracy and efficiency of diagnosis, such as fuzzy logic [17], support vector machine [18], extreme learning machine [19] and artificial neural networks [20].

However, the above fault-diagnosis methods of hydropower units are generally aimed at unidirectional vibration signals at one single measuring point. There is limited research on the axis orbit of hydropower units. Since the axis orbit displays the rotor’s running condition of the hydropower unit more intuitively, the identification of axis orbits provides another means for the fault diagnosis of the hydraulic turbine. The feature extraction and pattern recognition of the axis orbit are research areas for fault diagnosis for hydropower units. There are commonly used methods for feature extraction of the axis orbit, such as moment feature description [21], Fourier descriptor [22], and geometric parameter feature description [23], etc., which express the fault information of the axis orbit image in the form of a digital vector. Pattern recognition is mainly achieved by associating the extracted feature vector with the axis orbit type and then learning and training machine learning models such as artificial neural network (ANN) [24,25], support vector machine (SVM) [26], D-S evidence theory [27], etc., to realize the axis-orbit recognition. Chen et al. [24] proposed a method for identifying the axis orbit of hydropower units based on moment invariants and the BP neural network. Xue et al. [26] applied the fuzzy closeness feature of time series to extract the characteristic parameters of the axis orbit and optimized the SVM model for fault classification to improve the accuracy of hydraulic turbine diagnosis. Pang et al. [28] proposed a rotor system fault-identification method based on the axis orbit, integrating empirical mode decomposition (EEMD), morphological image processing, Hu invariant moment eigenvector, and the back-propagation (BP) neural network. The existing axis-orbit recognition methods have low recognition accuracy and poor robustness, while scholars primarily focus on the pattern recognition of axis orbits with single dimensions of feature set. Contour, moment, and geometric features describe the image shape from different dimensions. This paper combines these different dimensions of feature sets to improve the recognition accuracy of the axis orbit from the aspect of feature extraction. In addition, the RF-Fisher feature-selection method is used to decrease the feature dimensions, reducing redundant features to improve the efficiency of model operation. RF and Fisher are two classical feature-selection methods. The RF-Fisher method combines the advantages of good generalization performance, the high computational efficiency of Fisher [29], and the good robustness and information extraction capacity of RF [30], making the feature evaluation more scientific and objective. Moreover, as the parameter selection of SVM depends on a lot of experience and constant attempts, which consumes much time, GSA is used for optimizing the SVM model to adaptively select the optimal hyperparametric pair [c, g] of SVM while improving the recognition accuracy and generalization ability of the SVM model in this paper.

This paper proposes an axis-orbit recognition method based on feature combination and selection for the hydropower unit. First, the axis orbit images are preprocessed with morphological processing methods. Subsequently, the contour, moment, and geometric feature sets of the axis orbit samples are extracted, combined into a multidimensional feature set, and then selected by the RF-Fisher method. Finally, a trained GSA–SVM model is used as a classifier to recognize the patterns of axis orbits. The effects of different kinds of feature sets as inputs and the number of selected features on the recognition results are analyzed. In addition, the effectiveness and superiority of GSA–SVM as a classifier for axis-orbit recognition are verified compared to other state-of-the-art machine learning methods.

The rest of the paper is organized as follows. In Section 2, the basic theories of RF importance, GSA and SVM are introduced. Section 3 describes the proposed axis-orbit recognition method of the hydroelectric unit based on feature combination and feature selection. Then, the case study and result analysis are proposed in Section 4. Finally, conclusions are summarized in Section 5.

## 2. Theoretical Background

### 2.1. Random Forest Importance

Random Forest (RF) [30] takes decision trees as the basic unit, and each decision tree is a classifier. For an input sample, *K* trees will have *K* classification results. The Random Forest integrates all the classification results and takes the category with the most voting results as the final output.

Random Forests can be used to assess the importance of features. The main idea is to estimate how much each feature contributes to each tree in the Random Forest, then take the average value, and finally compare the contributions between features. The contribution is generally calculated by the Gini index or out-of-bag (OOB) data error rate [31].

### 2.2. Gravitational Search Algorithm

The gravitational search algorithm (GSA) [32] is a swarm intelligence optimization algorithm proposed by Esmat Rashedi et al. in 2009, based on the law of gravity and inter-particle interactions. It analogizes searching particles as several objects scattered in space and calculates the inter-particle interaction according to the universal gravitational force formula. In particle motion, the attraction is proportional to the mass, and the particle with the largest mass attracts other particles to move closer.

The gravitational force between particles and the resultant force of particles is calculated as Equation (1).

(1) $$\left\{ \begin{array}{l} F_{ij}^{k}(t)=G(t)\frac{M_{i}(t)\times M_{j}(t)}{R_{ij}(t)+\varepsilon }(x_{j}^{k}(t)-x_{i}^{k}(t))\\ F_{i}^{k}(t)=\sum_{j=1,j\neq }^{N}rand_{j}F_{ij}^{k}(t) \end{array}\right.$$

where, *ε*, *G*(*t*), *R*(*t*) denotes a very small number, the gravitational coefficient, and the spacing of particle *X_i_* and particle *X_j_*, respectively.

According to the Newton principle, the acceleration is defined as

(2) $$a_{i}^{k}(t)=\frac{F_{i}^{k}(t)}{M_{i}(t)}$$

The position and velocity of each particle are updated by Equations (3) and (4).

(3) $$v_{i}^{k}(t+1)=rand_{i}\times v_{i}^{k}(t)+a_{i}^{k}(t)$$

where, *a*(*t*) denotes acceleration of particle.

(4) $$x_{i}^{k}(t+1)=x_{i}^{k}(t)+v_{i}^{k}(t+1)$$

### 2.3. Support Vector Machine

The principle of support vector machine (SVM) [33] is to construct a model that converts a low-dimensional feature space into a high-dimensional feature space and find the optimal hyperplane, which separates various types of samples to achieve classification. Assuming a set of sample sets {*x_i_*, *y_i_*}, *i* = 1, 2, …, *m*, where *x_i_* is a feature vector consisting of n elements, and *y_i_* is the corresponding output of *x_i_*, the SVM maps to a high-dimensional space larger than *n* dimensions by a mapping function, the hyperplane can be constructed as Equation (5)

(5) $$f(x)=w^{T}\varphi (x)+b$$

where, *ω* and *b* denote the weight vector and the distance between the hyperplane and the origin, respectively.

The objective function and constraints for solving the optimal hyperplane are

(6) $$\left\{ \begin{array}{l} \min\frac{1}{2}{\left\|w\right\|}^{2}\\ y_{i}(w^{T}x_{i}+b)\geq 1,i=1,\cdots ,n \end{array}\right.$$

The solved optimal hyperplane is stated in Equation (7):

(7) $$\sum_{i=1}^{m}{\alpha_{i}}^{*}y_{i}(x_{j}\cdot x_{i})+b^{*}=0$$

## 3. The Proposed Method

**Figure 1:**
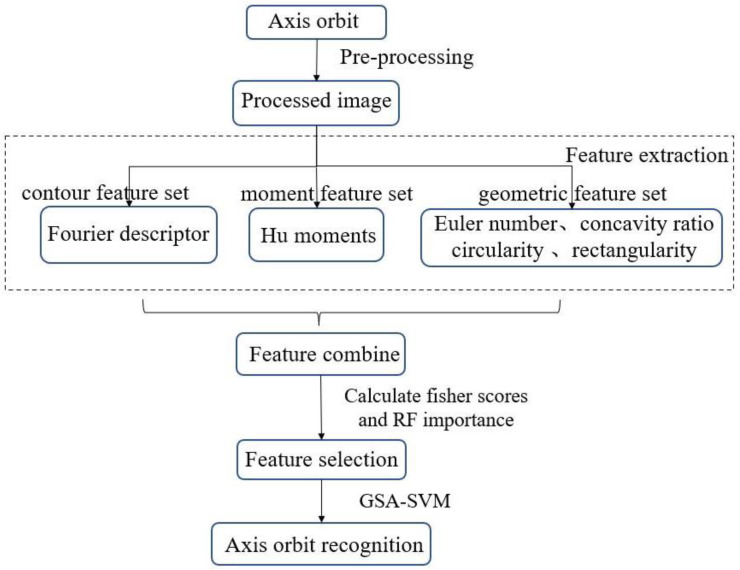
The flowchart of the proposed method.

In this work, a method for the axis-orbit recognition of hydroelectric units is proposed. Different kinds of features are combined as the input of the model, and GSA–SVM is applied as the classifier, aiming at the problems of low accuracy and the poor robustness of the existing axis-orbit recognition methods. In addition, the RF-Fisher feature-selection method is adopted to select top features to reduce the feature dimension, thus improving the efficiency of the model and reducing the runtime. The steps of the proposed method mainly include image preprocessing, feature extraction, feature combination and selection, and axis-orbit recognition. The flowchart is shown in Figure 1.

### 3.1. Axis Orbit Image Processing

Since vibration signals are disturbed by noise generally, the axis orbits become very chaotic. It is necessary to preprocess the axis orbit graph to eliminate the polluted noise and restore the actual axis orbit graph. The steps of image preprocessing include image binarization, multiple median filtering, skeletonization processing, pruning, and expansion. Firstly, the axis orbit image is binarized to reduce the amount of image data and highlight the target contour. Then, multiple median filtering is performed to reduce the noise in the image. The image is skeletonized after median filtering, and then some “burr” pixels in the image are removed by “pruning”. Finally, disc-shaped elements are used to expand the target.

### 3.2. Feature Extraction and Combination of Axis Orbit Samples

The axis-orbit recognition can be regarded as the pattern recognition of a 2D image. Generally, there are two types of description methods for image shape features: contour description and region description, which can be divided into geometric feature description and moment description. Fourier descriptors [21], Hu moment invariants [22] and Euler number, etc. [23], which are typical contour features, geometric features, and moment features, are extracted in this step.

The Fourier descriptor is based on the Fourier transform of the coordinate sequence of the shape contour boundary curve, which can be regarded as a frequency domain analysis of the closed boundary contour curve. The shape contour of the graph is considered as a closed curve formed by the motion of a moving point *s*(*t*) at its boundary as Equation (8).

(8) s(t)=x(t)+jy(t),t=0,1,2,…,T−1

Then *s*(*t*) is a periodic function, and the period is the length of the graph boundary. Converting *s*(*t*) into the form of Fourier series, the expansion coefficient *a*(*k*) of each term is the descriptor used to describe the axis orbit, defined in Equation (9).

(9) $$a_{k}=\sum_{t=0}^{T-1}s(t)\exp(-\frac{j2\pi kt}{T}),(0\leq t\leq T-1)$$

Set *a*(0) to zero and normalize the descriptor to obtain a compliant Fourier descriptor with translation, scaling, and rotation invariance, as stated in Equation (10).

(10) $$z(k)=\frac{\left\|a(k)\right\|}{\left\|a(1)\right\|},k=1,2,\ldots ,T-1$$

*z*(*k*) is the conforming Fourier descriptor.

The first 13 Fourier descriptors are selected as contour feature set *Fourier* = [*F*1, *F*2, …, *F*13].

Hu moment invariants are classical moment descriptors with translation, scaling, and rotation invariants constructed by Hu, which are constructed using the second- and third-order central moments under continuous image conditions.

For a digital image, its (*p + q*)th-order moments are defined as stated in Equation (11).

(11) $$M_{pq}=\sum_{x=0}^{M-1}\sum_{y=0}^{N-1}x^{p}y^{q}f(x,y)dxdy$$

The coordinates of the center of mass of a region in the image are

(12) $$\bar{x}=\frac{M_{10}}{M_{00}}\;\bar{y}=\frac{M_{01}}{M_{00}}$$

The (*p + q*)th-order central moment is defined in Equation (13).

(13) $$\mu_{pq}=\sum_{x=0}^{M-1}\sum_{y=0}^{N-1}f(x,y){\left(x-\bar{x}\right)}^{p}{\left(y-\bar{y}\right)}^{q}$$

The normalized central moment is

(14) $$\eta_{pq}=\frac{\mu_{pq}}{\mu_{00}^{r}}$$

where the *r* is subject to

(15) $$\gamma =\frac{p+q}{2}+1,p+q=2,3,\ldots$$

Hu moment invariants are as defined as stated in Equation (16).

(16) $$\left\{ \begin{array}{l} \phi_{1}=\eta_{20}+\eta_{02}\\ \phi_{2}={\left(\eta_{20}-\eta_{02}\right)}^{2}+4{\eta_{11}}^{2}\\ \phi_{3}={\left(\eta_{30}-3\eta_{12}\right)}^{2}+{\left(3\eta_{21}-\eta_{03}\right)}^{2}\\ \phi_{4}={\left(\eta_{30}+\eta_{12}\right)}^{2}+{\left(\eta_{21}+\eta_{03}\right)}^{2}\\ \phi_{5}=\left(\eta_{30}-3\eta_{12}\right)\left(\eta_{30}+\eta_{12}\right)[{\left(\eta_{30}+\eta_{12}\right)}^{2}-3{\left(\eta_{21}+\eta_{03}\right)}^{2}]+\\ \;\;\;\;\;\;\;\;\;\left(3\eta_{21}-\eta_{03}\right)\left(\eta_{21}+\eta_{03}\right)[3{\left(\eta_{30}+\eta_{12}\right)}^{2}-{\left(\eta_{21}+\eta_{03}\right)}^{2}]\\ \phi_{6}=\left(\eta_{20}-\eta_{02}\right)[{\left(\eta_{30}+\eta_{12}\right)}^{2}-{\left(\eta_{21}+\eta_{03}\right)}^{2}]+4\eta_{11}(\eta_{30}+\eta_{12})(\eta_{21}+\eta_{03})\\ \phi_{7}=\left(3\eta_{21}-\eta_{03}\right)\left(\eta_{30}+\eta_{12}\right)[{\left(\eta_{30}+\eta_{12}\right)}^{2}-3{\left(\eta_{21}+\eta_{03}\right)}^{2}]-\\ \;\;\;\;\;\;\;\;\;\left(\eta_{30}-3\eta_{12}\right)\left(\eta_{21}+\eta_{03}\right)[3{\left(\eta_{30}+\eta_{12}\right)}^{2}-{\left(\eta_{21}+\eta_{03}\right)}^{2}] \end{array}\right.$$

Hu moments of the axis orbit samples are calculated as the moment feature set *Hu* = [*Hu*1, *Hu*2, …, *Hu7*].

The four typical geometric features of the axis orbit samples are calculated to obtain the geometric feature set *Geom* = [*E, P, C, R*], which are Euler number, perimeter convexity ratio, rectangularity, and roundness.

Euler number is one of the important topological features in image analysis, which plays a vital role in image analysis and geometric object recognition. For 2D images, the Euler number is defined as stated in Equation (17).

(17) E=C−H

where, *C* and *H* denote the number of objects and holes, respectively.

The perimeter convexity ratio is used to measure the degree of concavity of the described area, as stated in Equation (18).

(18) $$Q=\frac{C_{0}}{C_{co}}$$

where, *C*_0_ and *C_co_* denote the perimeter of the target object and convex hull, respectively.

Rectangularity is the degree to which an object appears rectangular and is usually measured by the degree to which the object fills its outer rectangle., as defined in Equation (19).

(19) $$R=\frac{A_{o}}{A_{MER}}$$

where, *A*_0_ and *A_MER_* denote the area of the target object and its smallest outer rectangle, respectively.

Roundness, which describes the degree of roundness of a region, is measured by the ratio of the area to the square of the perimeter, as stated in Equation (20).

(20) $$F=\frac{4\pi A}{P^{2}}$$

where *A* and *P* denote the region area and the region boundary length, respectively. When the region is a circle, *F* = 1; when the region is other shapes, *F* < 1, and *F* with a relatively large value indicates that the shape of the region is closer to a circle.

The contour feature set, moment feature set, and geometric feature set are combined to obtain a multidimensional feature set *Features* = [*Fourier*, *Hu*, *Geom*].

### 3.3. Feature Selection

The RF importance and Fisher score [29] are used for feature evaluation in this step. The RF importance is calculated by the OOB data error rate in this paper. For each decision tree in the Random Forest, the corresponding out-of-bag data are used to calculate its out-of-bag data error, denoted as *errOOB1*. The number of decision trees is set to 20. Noise disturbance is randomly added to feature X of all samples included in the out-of-bag data, and its out-of-bag data error is calculated again, denoted as *errOOB2*. Supposing there are *N_tree_* trees in the Random Forest, the importance of feature X is

(21) $$importance=\frac{\sum \left(errOOB1-errOOB2\right)}{N_{tree}}$$

The RF OOB data error is calculated and ranked, and the RF importance of features is sorted as *Index*1 = [a1, a2, …, a24].

For classification problems, good features should have relatively similar values in the same category and distinctive values between categories. The Fisher score is obtained by calculating the distribution relationship between features and class variables, and the importance of feature *i* can be expressed by the Fisher score *S_i_* as

(22) $$S_{i}=\frac{\sum_{j=1}^{K}n_{j}{\left(\mu_{ij}-\mu_{i}\right)}^{2}}{\sum_{j=1}^{K}n_{j}{\rho_{ij}}^{2}}$$

where *μ_ij_* and *ρ_ij_* denote the mean and variance of feature *i* in category *j*, respectively, *μ_i_* and *η_j_* denote the mean of feature *i* and *η_j_* is the number of samples in category *j*, respectively.

According to Equation(21), important features always have significant inter-category differences and close intra-category distribution, thus possessing a high Fisher score. The Fisher scores of multidimensional features are calculated and sorted, and the Fisher scores of each feature are sorted as *Index*2 = [b1, b2, …, b24].

The features with Random Forest importance and Fisher score in the top n are selected as *Features_selection* = *index*1(1:n)∩*index2*(1:n).

### 3.4. Axis Orbit Recognition

The selected features are input into the trained GSA–SVM model for axis-orbit recognition. The RBF kernel function is selected for SVM in this paper, as stated in Equation (23).

(23) $$K(x_{i,}x_{j})=\exp(-g{\left\|x_{i}-y_{j}\right\|}^{2})$$

where *g* denotes kernel function radius.

The GSA is used to optimize the hyperparameter penalty coefficients and kernel function radius of SVM, and the recognition error rate is taken as the objective function, as defined in Equation (24).

(24) $$error=1-\frac{1}{n}\sum_{i=1}^{n}\left(y_{i}==\overset{\wedge }{y_{i}}\right)$$

The execution steps of GSA–SVM are as follows:

Step 1: Initialize the parameters of the algorithm. Set the population size N of individuals, the maximum number of iterations T, and randomly initialize the information about the location of individuals in space. N and T are set to 20 and 50, respectively.

Step 2: Calculate the magnitude of the fitness value for each object.

Step 3: Calculate the mass of each object.

(25) $$m_{i}(t)=\frac{fitness_{i}(t)-worst(t)}{best(t)-worst(t)}$$

where, *fitness*(*t*), *best*(*t*), and *worst*(*t*) denote the fitness value of particle X, the optimal solution, and the worst solution among all particles at moment t, respectively.

Step 4: Calculate the resultant of all other objects and the acceleration of each object according to Equations (1) and (2).

Step 5: Calculate the movement speed and update position information according to Equations (3) and (4).

Step 6: If the termination condition is not reached, skip to step 2 and iterate again; if the termination condition is reached, output the optimal solution.

## 4. Case study and Result Analysis

### 4.1. Data Description

The axis orbits of hydropower units come in various shapes under different fault conditions. Four typical unit faults and their corresponding axis orbit shapes are given in Table 1 [34].

It is difficult to obtain sufficient failure data due to the low failure probability during the operation of hydropower units. Numerical simulations for these four typical axis orbits were performed in a MATLAB environment according to Equation (26).

(26) $$\left\{ \begin{array}{l} x(t)=A_{1}\sin(\omega t+\alpha_{1})+A_{2}\sin(2\omega t+\alpha_{2})\\ y(t)=B_{1}\cos(\omega t+\beta_{1})+B_{2}\cos(2\omega t+\beta_{2}) \end{array}\right.$$

where *ω*, *A*_1_, *A*_2_, *a*_1_, *a*_2_, *B*_1_, *B*_2_, *β*_1_, *β*_2_ denote the angular velocity, the amplitude, and the initial phase of the fundamental and second harmonic in the *x*-direction and in the y direction, respectively.

**Figure 2:**
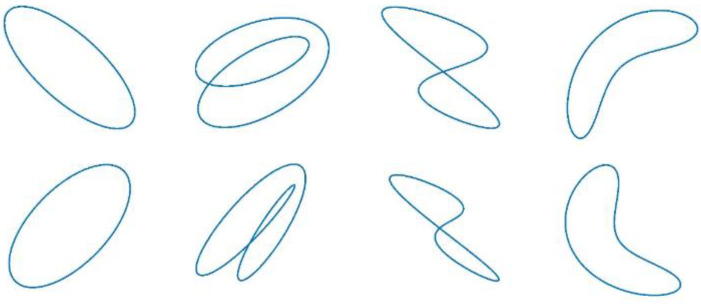
Generated axis orbit.

In our work, each of the elliptical-, outer 8-, inner 8-, and banana axis-type orbits were randomly generated 200 times using Equation (26) with different parameter values, and some of the generated axis orbit plots are shown in Figure 2.

In the actual industrial environment, the raw signal collected by the sensor contains background noise. In order to simulate the vibration signal in a realistic environment, the original signal was supplemented with Gaussian white noise, as stated in Equation (27).

(27) $$\left\{ \begin{array}{l} x^{\prime }(t)=x(t)+x\_noise\\ y^{\prime }(t)=y(t)+y\_noise \end{array}\right.$$

The signal-to-noise ratio (SNR) equation is stated in Equation (28).

(28) $$SNR=10\log_{10}\frac{P_{s}}{P_{n}}$$

where *P_s_* and *P_n_* denote the signal power and the noise power, respectively.

**Figure 3:**
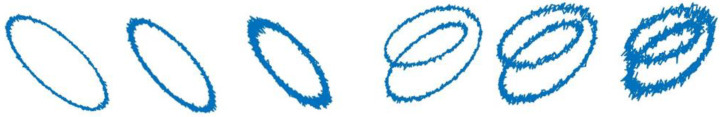

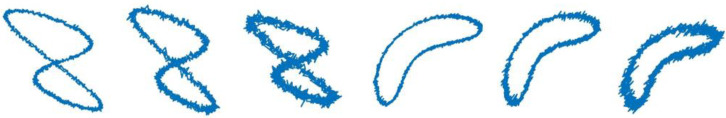
The axis orbits with noise.

We added noise with SNR values of 30, 25, and 20, respectively, and the obtained axis orbits are depicted in Figure 3.

### 4.2. Data Processing

**Figure 4:**
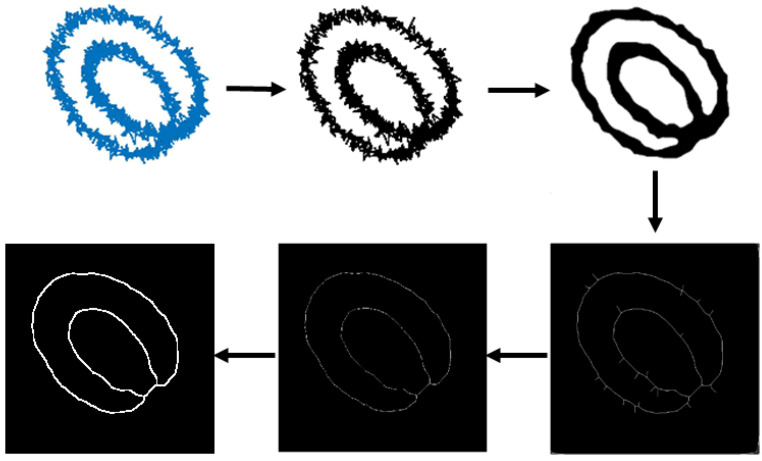
Preprocessing of axis orbit image.

First, the axis orbit images were pre-processed. The intermediate images of the axis orbit image following a series of data processing—image binarization, multiple median filtering, skeletonization, pruning, and expansion—are illustrated in Figure 4.

It can be seen from Figure 4 that the purpose of the purification filtering was preliminarily achieved after pre-processing.

**Figure 5:**
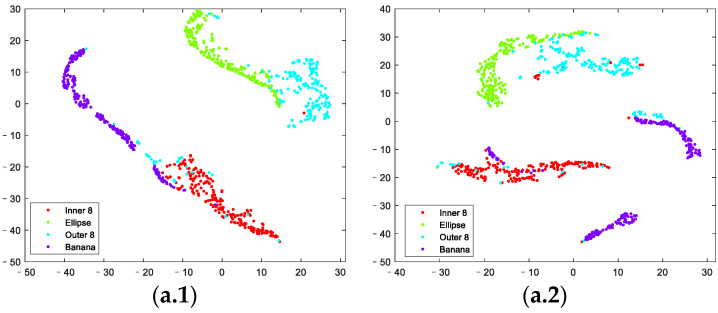

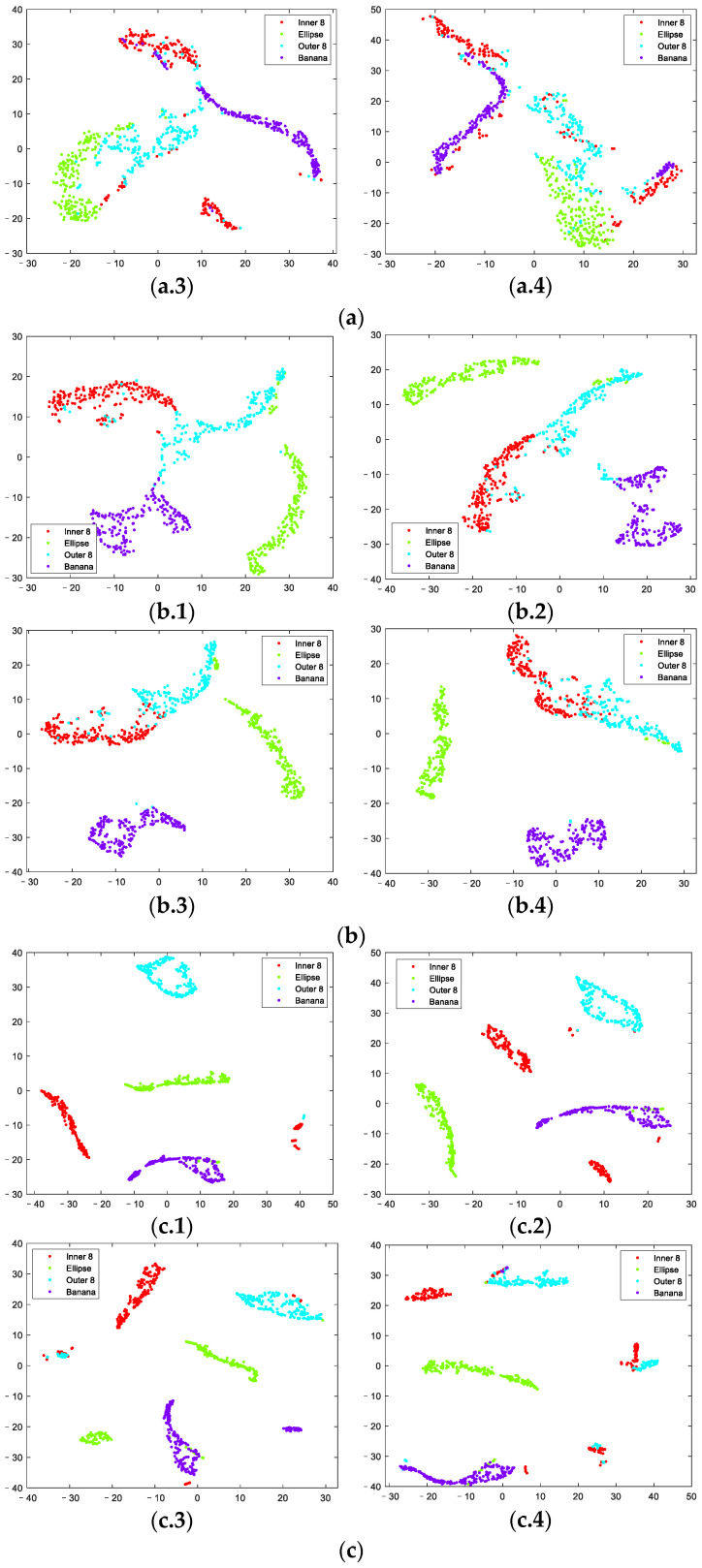
T-SNE of each feature set. (**a**) contour feature set: (**a.1**) axis orbit with no noise. (**a.2**) axis orbit with noise of SNR30. (**a.3**) axis orbit with noise of SNR25. (**a.4**) axis orbit with noise of SNR20. (**b**) moment feature set: (**b.1**) axis orbit with no noise. (**b.2**) axis orbit with noise of SNR30. (**b.3**) axis orbit with noise of SNR25. (**b.4**) axis orbit with noise of SNR20. (**c**) geometric feature set: (**c.1**) axis orbit with no noise. (**c.2**) axis orbit with noise of SNR30. (**c.3**) axis orbit with noise of SNR25. (**c.4**) axis orbit with noise of SNR20.

Then, the contour feature set, moment feature set, and geometric feature set of the axis orbit samples, containing 13, 7, and 4 features of each axis orbit sample, respectively, were calculated according to step (2) in Section 3. The visualizations of the extracted contour feature set, moment feature set, and geometric feature set of the axis orbit samples with different noise contents in a two-dimensional feature space using t-Distributed Stochastic Neighbor Embedding (T-SNE) are displayed in Figure 5.

In this study, the contour feature set, moment feature set, and geometric feature set were merged to obtain the multidimensional feature set of the axis orbit samples.

### 4.3. Analysis of Results

The experiment was conducted for four kinds of axis orbit sample statuses, i.e., samples without noise, samples with the noise of *SNR* = 30, samples with the noise of *SNR* = 25, and samples with the noise of *SNR* = 20, respectively. For each ellipse-, outer 8-, inner 8- and banana-shaped axis orbit image, 150 out of 200 samples were randomly selected as the training samples under each status, and the remaining 50 were set as the test samples.

#### 4.3.1. Recognition Result with Various Feature Sets

**Figure 6:**
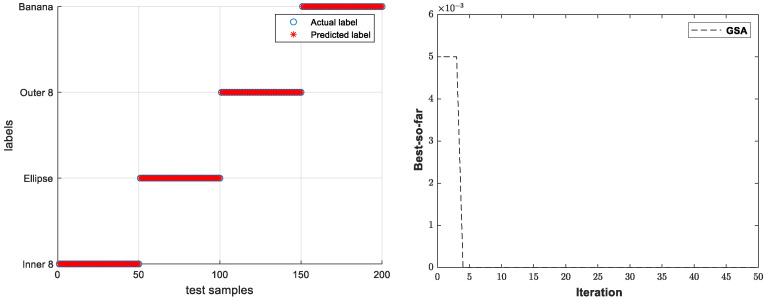
The identification results and the iteration process of GSA.

The contour feature set, moment feature set, geometric feature set, and multi-dimensional feature set were chosen as the input of GSA–SVM for axis-orbit recognition. The identification results and the iteration process of GSA of one of the experiments are shown in Figure 6.

We performed the experiment 50 times under each test condition and took the average value as the axis-orbit recognition accuracy. The statistical results are shown in Table 2.

From Table 2, it can be found that the recognition accuracy of the multidimensional feature set as the model input is the highest among all these feature sets for axis orbit samples with various noise contents. As the noise amplitude increases, the accuracy falls most smoothly when taking the multidimensional feature set as the input. It indicates that the model achieves higher recognition accuracy and better robustness behavior by taking the multidimensional feature set as the input than just taking a single kind of feature set as the input.

**Figure 7:**
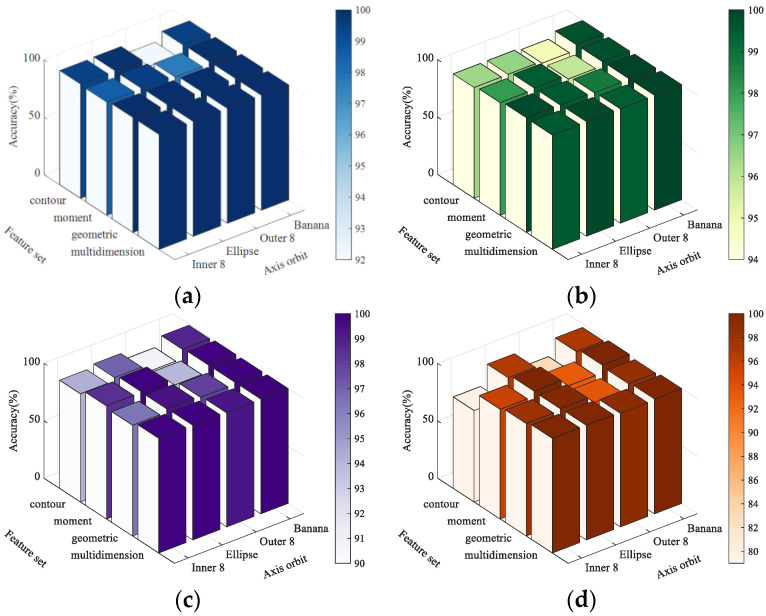
Recognition results of each type of axis orbit. (**a**) axis orbit with no noise. (**b**) axis orbit with noise of SNR30. (**c**) axis orbit with noise of SNR25. (**d**) axis orbit with noise of SNR20.

In addition, we calculated the recognition accuracy of each type of axis orbit while various types of feature sets were used as the input, and the results are shown in Figure 7. Figure 7 shows that taking the multidimensional feature set as the input results in a smaller standard deviation of the recognition accuracy than taking any single type of feature set as the input, for every axis orbit sampling set, which indicates that using multidimensional features as the input enables the model to identify various axis orbits more robustly.

#### 4.3.2. Comparison Experiments

**Figure 8:**
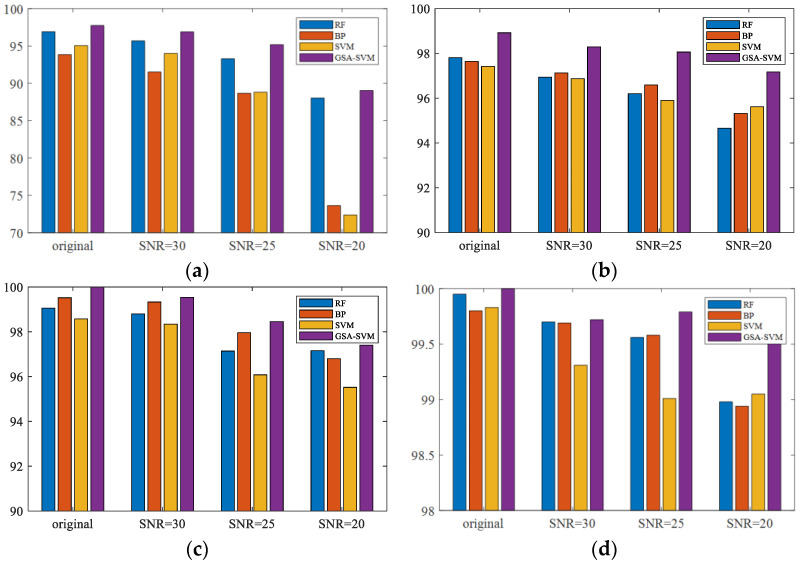
Recognition results with different classifiers. (**a**) merge contour feature set as input. (**b**) moment feature set as input. (**c**) geometric feature set as input. (**d**) multidimensional feature set as input.

In this sub-subsection, Random Forest, setting SVM, and the BP Neural Network were selected as the intelligent classifiers for comparative experiments. We took the average value of 50 trials as the results for each algorithm; the experimental results are given in Table 3, and the corresponding statistical charts are presented in Figure 8. The results show that regardless of either classifier, the recognition accuracy with the multidimensional feature set as the input is higher than that with any single feature set as the input, which is consistent with the conclusions in Section 4.3.1. Moreover, the recognition accuracy of the proposed GSA–SVM used in this paper is higher than that of other machine learning methods as classifiers under various experimental conditions. The recognition accuracy of GSA–SVM possesses the smallest fluctuation aptitude when the noise proportion or the feature set input changes.

#### 4.3.3. Feature Selection

**Figure 9:**
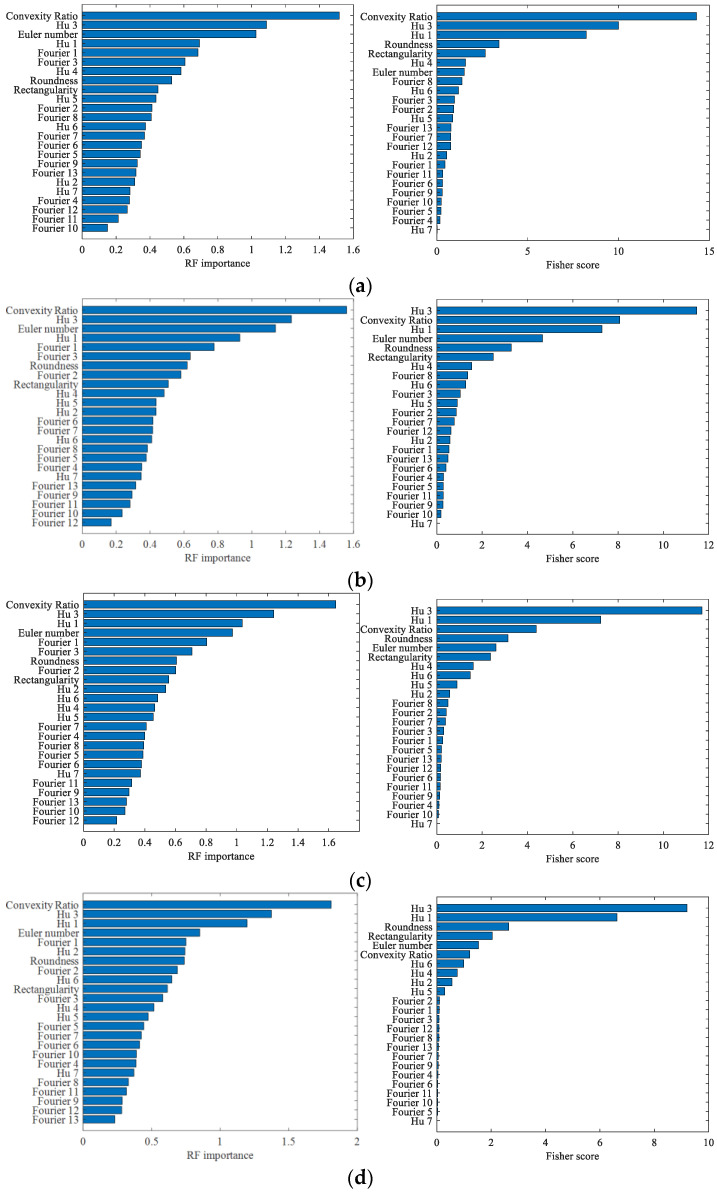
Feature score ranking. (**a**) axis orbit with no noise. (**b**) axis orbit with noise of SNR30. (**c**) axis orbit with noise of SNR25. (**d**) axis orbit with noise of SNR20.

In this sub-subsection, we performed feature selection with the Fisher-RF algorithm. The Random Forest importance and Fisher score of features were calculated and ranked to obtain the ranking chart of each feature, as shown in Figure 9.

**Figure 10:**
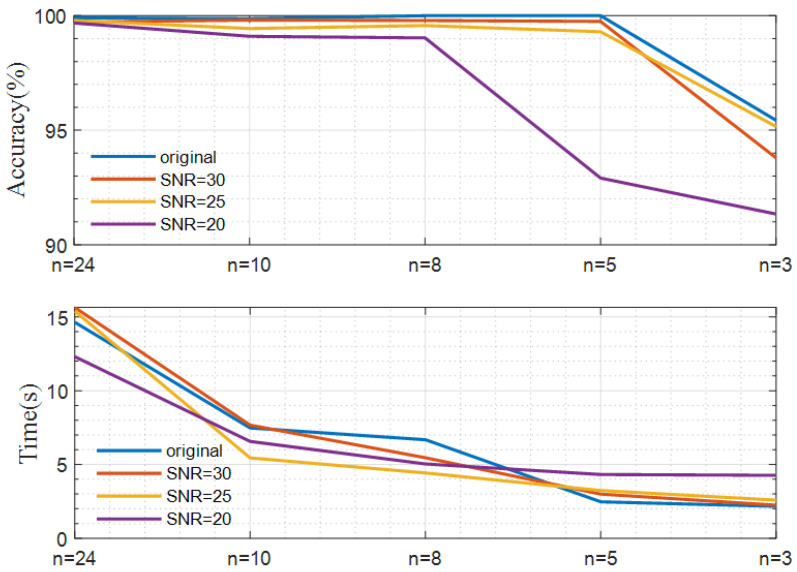
Recognition accuracy and time with different n sets.

As for a large feature set, there may be uncorrelated, redundant, and non-differentiable features, which leads to a longer time for analyzing features and training models. Removing redundant features and selecting an effective feature subset can lower the data complexity and reduce the time required for model operation. This paper selects the features ranking in the top *n,* with the two feature evaluation indicators as the input of the GSA–SVM model, and sets different *n* values for tests. The accuracy of the model and the time required under different *n* settings are compared to select the most compelling feature subset, reducing the training time of the model under the condition of ensuring the accuracy of the model as much as possible. The experimental results are shown in Table 4 and presented in Figure 10.

From Figure 10 and Table 4, it can be seen that the recognition accuracy and the model running time both tend to decrease when *n* becomes smaller. The appropriate setting of *n* ensures sufficient recognition accuracy and an affordable running-time cost. For axis orbit samples without noise, samples with the noise of SNR = 30, and samples with the noise of SNR = 25, the recognition accuracy does not decrease significantly, while the model operation efficiency is greatly improved with an appropriate choice of *n* = 5, and we can also find that *n* = 8 is suitable for the axis orbit samples with the noise of SNR = 20.

In order to test the performance of the feature-selection algorithm (RF-Fisher) used in this paper, mRMR and laplacion, two general feature-selection algorithms were selected for comparison with RF-Fisher, and the GSA–SVM algorithm was used for training. The above 3 algorithms were tested 50 times and their mean values were obtained, as shown in Table 5.

It can be seen from the table that when the selected feature number decreases, the RF-Fisher method maintains higher recognition accuracy compared with mRMR and laplacian, which indicates that under the unified accuracy requirements, RF-Fisher has better performance in feature dimension reduction. The effectiveness and superiority of the RF-Fisher feature-selection method are verified.

## 5. Conclusions

An axis-orbit recognition method based on feature combination and feature selection is proposed in this paper. The data analysis results verify the effectiveness of the proposed scheme and its superiority over the existing methods. The following conclusions can be drawn:

(a) When multidimensional features are used as the model input, the recognition accuracy of axis orbit is higher than that of the single category feature, which indicates that multidimensional features can improve the recognition accuracy of axis orbits from the feature aspect, compared with a single kind of feature;

(b) Compared with RF, SVM, and the BP neural network, GSA–SVM has higher recognition accuracy and better robustness behavior, which shows the superiority of the GSA–SVM method when applied to axis-orbit recognition;

(c) Feature selection based on RF-Fisher can reduce the running-time cost of the proposed model while ensuring the accuracy of the model to a certain extent.

Due to the lack of real fault cases and relevant data, this paper verifies the effectiveness of the proposed method with the simulated standard axis orbit graphs. In engineering practice, the axis orbits of different hydropower units are not the same in their characteristics. The further accumulation and verification of cases and the application of the proposed method to actual hydropower units is the next step to be studied.

## Figures and Tables

**Table 1:** Typical axis orbit of hydropower units.

Fault Status	Imbalance	Oil-Film Whirl	Misalignment	Imbalance and Misalignment
Axis orbit	Ellipse	Outer 8	Banana	Inner 8

**Table 2:** GSA–SVM recognition results with different feature set inputs.

	Feature Set	Merge Contour	Moment	Geometric	Multidimensional
Axis Orbit					
Original		100	100	99.32	99.83
SNR_30		96.89	98.29	99.54	99.72
SNR_25		95.17	98.06	98.45	99.79
SNR_20		89.04	97.17	97.4	99.68

**Table 3:** Recognition results with different classifiers.

Axis Orbit	Classifier	Accuracy(%)			
		Contour	Moment	Geometric	Multidimensional
Original	RF	96.92	97.81	99.06	99.95
	BP	93.85	97.64	99.52	99.8
	SVM	95.06	97.42	98.58	99.83
	GSA–SVM	97.74	98.92	100	100
*SNR* = 30	RF	95.69	96.94	98.8	99.7
	BP	91.52	97.13	99.33	99.69
	SVM	94.01	96.87	98.34	99.31
	GSA–SVM	96.89	98.29	99.54	99.72
*SNR* = 25	RF	93.3	96.2	97.14	99.56
	BP	88.67	96.59	97.96	99.58
	SVM	88.82	95.9	96.08	99.01
	GSA–SVM	95.17	98.06	98.45	99.79
*SNR* = 20	RF	88.04	94.66	97.16	98.98
	BP	73.64	95.32	96.8	98.94
	SVM	72.38	95.62	95.52	99.05
	GSA–SVM	89.04	97.17	97.4	99.68

**Table 4:** Recognition accuracy and the time required under different n sets.

	Original		SNR30		SNR25		SNR20	
	Accuracy (%)	Time (s)	Accuracy (%)	Time (s)	Accuracy (%)	Time(s)	Accuracy (%)	Time (s)
*n* = 24	99.91	14.637	99.72	15.6380	99.79	15.3885	99.68	12.3085
*n* = 10	99.87	7.4758	99.81	7.6680	99.44	5.4476	99.1	6.5624
*n* = 8	100	6.6776	99.79	5.4589	99.57	4.4267	99.03	5.0380
*n* = 5	100	2.4714	99.75	2.9931	99.3	3.2378	92.91	4.3200
*n* = 3	95.43	2.1678	93.79	2.2439	95.16	2.5835	91.34	4.2669

**Table 5:** Recognition results with different feature-selection methods.

Data Set	Method	Recognition Accuracy with Different Selected Feature Number							
		10	8	7	6	5	4	3	2
original	mRMR	99.89	99.88	99.91	99.94	99.13	98.87	96.78	95.53
	laplacian	99.92	99.85	99.06	95.89	95.9	93.12	92.72	92.18
	RF-Fisher	--	99.87	--	100	--	--	100	95.43
SNR30	mRMR	99.63	99.63	99.61	99.62	99.61	98.52	96.72	94.23
	laplacian	99.85	99.81	98.97	95.06	94.89	92.53	92.13	91.63
	RF-Fisher	--	99.81	--	--	99.79	99.75	--	93.79
SNR25	mRMR	99.77	99.92	99.95	99.91	97.7	97.46	96.94	94.66
	laplacian	99.82	99.41	99.38	98.84	94.04	94.71	94.78	90.86
	RF-Fisher	--	--	99.44	--	99.57	99.3	--	95.16
SNR20	mRMR	99.3	99.26	99.25	99.36	98.48	94.44	94.2	91.52
	laplacian	99.74	96.75	96.93	96.62	96.17	96.03	93.28	87.31
	RF-Fisher	--	99.1	--	--	99.03	--	92.91	91.34

## Data Availability

No new data were created.
